# Hepatitis B infection and risk factors among children living with HIV in Yaounde, Cameroon: an integrated management

**DOI:** 10.1186/s12887-019-1750-x

**Published:** 2019-10-22

**Authors:** Fokam Joseph, Kamga Wouambo Rodrigue, Tchatchouang Serges, Nguwoh Philippe Salomon, Taheu Ngounouh Christian, Tommo Tchouaket Michel Carlos, Fosso Samuel, Njom-Nlend Anne-Esther, Vittorio Colizzi, Nkenfou Nguefeu Celine

**Affiliations:** 1 Chantal BIYA International Reference Centre (CIRCB) for Research on HIV/AIDS prevention and management, Yaounde, Cameroon; 2 Faculty of Medicine and Biomedical Sciences of the University of Yaounde 1, Yaounde, Cameroon; 3 National HIV Drug Resistance Working Group, Ministry of Public Health, Yaounde, Cameroon; 4 Faculty of Science, Department of Microbiology and Parasitology, University of Buea, Buea, Cameroon; 5 Department of Health Sciences, Estuary Academy and Strategic Institute (IUES/INSAM/ISSAS), Higher Institute of Health Applied Sciences, University of Buea, Buea, Cameroon; 6 Faculty of Science, Department of Biochemistry, University of Yaounde 1, Yaounde, Cameroon; 7 National Public Health Laboratory, Ministry of Public Health, Yaounde, Cameroon; 8 Higher Institute of Sciences and Techniques Applied to Health (ISTASS), Yaounde, Cameroon; 9 Laboratoire Biosanté International, Yaounde, Cameroon; 10 Essos Hospital Centre (CHE), Yaounde, Cameroon; 11 Cameroon Evangelic University, Bandjoun, Cameroon; 12 UNESCO Board of Biotechnology, University of Rome Tor Vergata, Rome, Italy; 13 Higher Teacher Training College (ENS), University of Yaounde 1, Yaounde, Cameroon

**Keywords:** HBV infection, Risk factors, Children living with HIV/AIDS

## Abstract

**Background:**

The endemicity of hepatitis B virus (HBV) prompted the systematic immunization of newborns in Cameroon since 2005. In the frame of a considerable burden of HIV/HBV co-infection (17.5%), monitoring HBV among children living with HIV (CLHIV) would guide toward HIV/HBV integrated paediatric care. We sought to ascertain the prevalence and determinants of HBV infection in the population of CLHIV and performance of commonly used rapid diagnosis tests (RDTs).

**Methods:**

Cross-sectional study conducted from February through June 2017 in a subset of CLHIV ≤15 years old at the Essos Hospital Centre, Yaounde, Cameroon. HBV was tested by HBsAg ELISA sandwich in duplicates for each sample, and the mean optical density was calculated. The Determinants of HBV-prevalencewere evaluated, and *p* < 0.05 was the significance threshold. The performance of two HBV RDTs (Diaspot vs. HBV-5) was evaluated in comparison to ELISA (used as gold standard).

**Results:**

Of the 83 CLHIV enrolled (54.2% female, mean age 8.7 [±3.8] years, 60% vaccinated against HBV, all breastfed), HBV-prevalence was 2.41% (2/83). HBV-positivity was significantly associated with unknown maternal HBV status (2.9% [2/69] vs. 0.0% [0/14], *p* = 0.0097) and vaginal delivery (2.4% [2/82] vs. 0.0% [0/1], *p* = 0.0018). Moreover, the most likely to be positive were aged 11 and 15 years, and had experienced neither anti-HBV vaccination nor anti-HBV serum administration, and both had not been treated with any antiseptic solution at birth. Regarding the performance of Diaspot vs. HBV-5 respectively, sensitivity was 100% (2/2) vs. 50% (1/2), while specificity was 100% (45/45) vs. 97.8% (44/45); positive and negative predictive values of Diaspot versus HBV-5 were respectively 100% (2/2) and 100% (45/45) versus 50% (1/2) and 97.8% (44/45).

**Conclusion:**

HBV-infection in the population of CLHIV appears at a moderate prevalence, suggesting a decreased burden likely due to preventive measures including the wide vaccine coverage. Focusing on mothers with unknown HBV status and promoting safer delivery mode (caesarean section) for HBV-positive motherswould contribute toward pediatric HBV elimination. In context of limited resources, Diaspot test appears more reliable to rollout HBV-infection in the population of CLHIV. As findings are limited to a small sample size, studies on a wider population would be relevant.

## Introduction

HIV and hepatitis B virus (HBV) remain major public health concerns worldwide, with about 36.9 and 350 million people living respectively with HIV and chronic HBV (CHB) infections worldwide [1, 2]. While 70% of the global HIV burden is found in sub-Saharan Africa (SSA), HBV is ubiquitous within the Asian (15%) and African populations (8–40%) [2], thus indicating a high risk of HIV/HBV co-presence within the African populations. Of note, HIV/HBV co-infection is characterized by a synergistic interaction between both viruses leading on one hand, to a rapid progression to AIDS defining event(s), and on the other hand, to an earlier development of hepatocellular carcinoma as compared to HBV mono-infected individuals [3]. Also, the probability of acquiring CHB is significantly higher in the frame of HIV infection, and this event becomes more worrisome for children at early age (80–90% of infants aged below 1 year and 30–50% of children aged 1–4 years) infected with HBV end-up developing CHB infection [4, 5]. Thus, in order to support the global effort of eliminating viral hepatitis, strategies toward integrated prevention and care for HIV/HBV would be essential, especially for paediatric populations for whom the risk of CHB is the highest [2, 3].

The common routes of HBV transmission among children are perinatal and horizontal during early childhood [6, 7]. Of note, perinatal transmission was more frequent in Asia (from mother-to-child) while the horizontal was more in SSA (horizontally from infected household members or unsafe materials during early age) in year 1990 [8, 9]. Of relevance, “children detected positive for hepatitis B surface antigen (HBsAg) and envelope antigen (HBeAg) are highly contagious; over 85–90% of them are potential CHB carriers and liver cancer patients at adulthood, a condition that would be worsening in the context of co-infection with HIV [10–12]. In this context, ensuring an effective implementation of a preventive package would be helpful, starting from universal screening of HBV among pregnant women attending their first antenatal care (ANC), a systematic vaccination against HBV all pregnant women having a negative HBsAg result, providing HBV antibodies at birth to all vertically-exposed babies, and a universal vaccination of new-borns [13]. However, response to anti-HBV vaccine might vary according to age and HIV status, thereby indicating high vulnerability to HBV among children living with HIV (CLHIV) as we previously reported within a similar target population in Cameroon [13]. Thus, assessing the burden of HBV in CLHIV in Cameroon would help in understanding the current epidemiologic context and in designing interventions for greater protectiveness at country-level.

Even though Cameroon is experiencing declining trends of both HIV among adults and adolescents aged 15–49 years (from 4.3 to 3.4%) and HBV (12.2 to 8.1%), the country is still experiencing a generalized epidemic of both infections, suggesting consistent risks of paediatric infections and the need for evidence-based strategic interventions to support an integrated care [14, 15]. Though data on HBV/HIV coinfection in children remain largely uncovered in several SSA countries, our previous findings from Cameroon indicate a poor immunological response to HBV-vaccine in the population of CLHIV, hence suggesting risks of HBV-infection in spite of vaccination [13]. Additionally, HBV/HIV prevalence remains high in several African countries (5–40%), with 17.5% coinfection among pregnant women in Cameroon [7, 16–18] where the screening of HBV in new born babies is not yet systematically done. Furthermore, antiretroviral therapy (ART), for both children and adults living in SSA, usually entails molecules with anti-HBV activities (lamivudine [3TC], emtricitabine [FTC], tenofovir [TDF]), with limited evidence on its effects on HBV pathogenesis in the frame of co-infection with HIV in children [16, 17]. Thus, understanding factors sustaining risks of HBV transmission to CLHIV is crucial for designing integrated but impactful strategies against HBV for CLHIV in SSA [16, 17].

Integrating HBV service delivery with HIV care requires the use of simple, affordable and rapid diagnostic tests (RDTs) for HBV screening in SSA settings [7, 13]. Of note, in spite of their high sensitivity and specificity, reference diagnostic assays remain costly, require sophisticated platforms and are time consuming [13]. In this frame, RDTs are highly encouraged and are widely used for HBV screening, but without a prior assessment of the diagnostic accuracy for ensuring clinical validation of result delivered locally [13]. Thus, assessing the performance of commonly used HBV RDTs in routine practice would prompt the rollout of HBV/HIV service integration and support the global effort toward elimination, especially in children known to have a higher risk of vulnerability to infection and rapid disease progression if co-infected.

The objectives of our study were to evaluate the prevalence of HBV in the population of CLHIV, determine factors associated with HBV-infection in these children, and assess the diagnostic performance of two RDTs routinely used for HBV detection.

## Materials and methods

### Study design and settings

A hospital-based study targeting CLHIV visiting the HIV Care and Treatment Centre of the Essos Hospital Center (EHC) in Yaounde-Cameroon was conducted from February 3rd to June 30th, 2017. EHC was selected as the study sentinel site based on the availability of a paediatric care service, the availability of standard registers for monitoring individuals diagnosed HIV-positive, the management of ART following the national guidelines, and a cohort of CLHIV receiving ART on site. In fact, HIV testing is systematically performed across the country on each pregnant woman and each child vertically exposed to HIV unlike HBV testing, not yet widespread and consequently just done at times on pregnant women, children exposed infected or non-infected.

### Sample size calculation

All eligible children visiting the study site were enrolled consecutively during the 5 months study period. Using an estimated HBV prevalence of 8.1% in HIV-infected Cameroonians from the CAMPHIA survey [15], a Z-score at 95% confidence interval (Z = 1.96), and an error rate of 6%, the following formula was used to calculate the sample size (*N* = 80 participants):

$$ \mathrm{N}\ \left(\mathrm{size}\right)={Z}^2\mathrm{x}\ \frac{\mathrm{P}\left(1-\mathrm{P}\right)}{{\left(\mathrm{i}\right)}^2}=79.4 $$

### Enrolment of participants

Following a consecutive sampling strategy, study participants were enrolled during their routine clinic attendance at the study site (EHC). Inclusion criteria were: (a) be HIV-positive; (b) be aged ≤15 years old; (c) have an unknown HBV status; (d) be registered for monitoring at the study site; (d) not being transferred from another health facility; (e) have maternal/guardian informed consent for participation. Any child fulfilling the aforementioned criteria was excluded if unable to provide a blood sample.

#### Data collection

After consenting, a standard questionnaire was administered to the respective mother or guardian of eligible CLHIV, covering data on socio-demographic information (sex, age, region, origin, mother-child HBV risk factors) and epidemiological and clinical information, ART history, adherence information during pregnancy, delivery mode, infant feeding option, knowledge of maternal HBV status, maternal and child HBV vaccination history, possible exposure of the child to HBV, etc. For participants aged 11 years and above with high risk to suxual active, the questionnaire was filled, following by an interview in order to get more information on their sexual behaviours and others factors exposing to HBV in relation with their age range. Data were also verified and completed using the medical records available at the health facility.

#### HBV testing

A total of 5 mL of whole blood was collected in dry tubes by venipuncture from each study participant; after centrifugation, serum samples were separated from whole blood and stored at − 20 °C. Serum samples were then tested for HBV serological testing at the BIOSANTE INTERNATIONAL laboratory located close to the clinical site (EHC) in Yaounde, Cameroon.

For the evaluation of HBV prevalence, HBV serological testing targeting HBsAg was performed in duplicates using enzyme immuno-assay as per manufacturer’s instructions (EIA-HBsAg test Kit-Rapid Labs, United Kingdom). Results were interpreted by calculating the mean of optical density of the duplicates with reference to the cut-off values as per the manufacturer’s interpretation. According to the manufacturer, invalid results were specimens with absorbance to cut-off ratio between 0.9 and 1.1 and imposing another retesting of these specimens in duplicates instantaneously or repeat the test one month later.

For evaluation of the diagnostic performance of RDTs, the two lateral flow immuno-chromatographic assays for the detection of HBsAg, HBSAg DiaSpot®rapid test and HBV 5 in 1 kit (Hepatitis B 5 markers in one rapid diagnosis test), were tested on each sample as per the manufacturer’s instructions. Results were reported either as positive, negative or invalid. Using the EIA (the gold standard), a technic routinely used as the confirmation test of HBV infection,. Sensitivity, specificity, positive predictive value (PPV) and negative predictive values (NPV) were calculated as previously described by Marcillat et al [19].

### Statistical analysis

Data were entered into an excel spreadsheet, double-checked for accuracy and cleaning, then closed for data analysis. The cleaned dataset was then transferred into Epi info 7.0 for statistical analyses. HBV prevalence was defined as low if < 2%, moderate if between 2 and 7%and high if ≥ 8[20]. The comparison of categorical variable was done using a X^2^ test or Fisher exact test wherever applicable, and *p*-value < 0.05 was considered as statistically significant.

## Results

### Characteristics of study participants

A total of 83 eligible participants were finally included in the study. The sex distribution was slightly different (54.2% female vs. 45.80% male); the mean age was 8.7 (±3.8) years and distributed as follows: 15.7% (13/83) aged [0–5[, 36.1% (30/83) aged [5–10[and 48.2% aged [10–15] years (Table 1).

**Table 1:** Sociodemographic and basic clinical data of the study population

Overall study participants	83 (100%)
CD4 cells count, cells/mm^3^, median [IQR]	1031 [830–1330]
VL	
< 50	21 (25.3%)
50–999	13 (15.6%)
≥ 1000	15 (18.1%)
Unknown viremia	34 (41%)
WHO clinical Stages, n(%)	
I	72 (86.7%)
II	6 (07.2%)
III	3 (3.6%)
IV	2 (2.4%)
Gender, n(%)	
Male	38 (45.8%)
Female	45 (54.2%)
Age (Year), Median [IQR[	9 [6–12]
Preterm babies	
Yes	5 (6.0%)
Non	78 (94.0%)
Feeding option	
Exclusive breastfeeding	24 (28.9%)
Replacement feeding	18 (21.7%)
Mixed feeding	41 (49.4%)
Infant anti-HBV vaccination status	
Yes	50 (60.3%)
No	33 (39.7%)
Bath at birth	
Yes	21 (25.3%)
No	62 (74.7%)
Maternal HAART during pregnancy	
TDF-3TC-EFV	64 (77.1%)
Other regimens	13 (15.7%)
ART-Naïve	6 (7.2%)
Maternal anti-HBV vaccination status during pregnancy	
Negative	08 (9.6%)
Positive	01 (1.2%)
Unknown	69 (83.1%)
Vaccinated	05 (6.02%)

*ART* antiretroviral therapy, *HAART* Highly Active Antiretroviral Therapy, *HAART* Highly Active Antiretroviral Therapy, *HBV* hepatitis B virus, *IQR* interquartile range, *TDF-3TC-EFV* tenofvoir-lamivudine-efavirenz, *WHO* World Health Organisation

Regarding maternal history of HBV during pregnancy, 83.1% (69/83) had no knowledge of their HBV status vs. 16.9% (who already had documented HBV results), of whom only one was reported HBsAg positive. Regarding HBV-vaccine, only five mothers (6.0%) declared to have received anti-HBV vaccine. Regarding ART exposure, 7.2% declared to be ART-naïve during pregnancy vs. 92.8% ART-experienced mothers receiving either tenofovir-lamivudine-efavirenz (TENLAM-E) or other ART regimens (Table 1).

Based on children’s immunization records, 60.2% (50/83) had a complete history of anti-HBV vaccination as per the national guidelines of the immunization program in Cameroon. None of the participants was sexually active (as per individual reports) and 77.1% (64/83) did not have any history of blood transfusion (as per data from medical records) (Table 1).

### HBV prevalence

The prevalence HBV, defined as the presence of HBsAg, was 2.4% (2/83) in the entire study population, indicating a moderateprevalence of HBV. The two HBV-positive cases were both female (aged 10–15 years), without any statistically significant difference as compared to males: 4.4% (2/45) vs. 0.0% (0/38), *p* = 0.498.

### Risk factors of HBV among study participants

According to delivery mode, children borne by vaginal delivery were more likely to be infected with HBV as compared to those born by caesarean section: 2.4% vs. 0% respectively, *p* = 0.0018 (Table 2).

**Table 2:** Presence of HBsAg according to potential exposure

Exposure		N	Positivity rate of HBsAg	*p*- value
Preterm baby	Yes	5	0	*p* = 0.25 X^2^ = 1.3
	No	78	2 (2.6%)	
Feeding option	Exclusive breastfeeding	24	2 (8.3%)	*p* = 0.41 X^2^ = 1.76
	Replacement feeding	18	00	
	Mixed feeding	41	00	
Antiseptic bath at birth	Yes	21	00	*p* = 1 X^2^ = 00
	No	62	2 (3.2%)	
Infant anti-HBV vaccination status	Yes	50	00	*p* = 0.3 X^2^ = 1.063
	No	33	02 (6.1%)	
Maternal HBV status during pregnancy	Negative	08	00	***p = 0.0097*** **X**^**2**^ **= 11.374**
	Positive	01	00	
	Unknown	69	**02 (2.9%)**	
	Vaccinated	05	00	
Delivery mode	Normal vaginal	82	**02 (2.4%)**	***p = 0.0018*** **X**^**2**^ **= 9.75**
	Caesarean section	01	00	
Gravidity	Primiparous	19	00	*p* = 0.94 X^2^ = 0.005
	Multiparous	64	02 (3.1%)	
Maternal HAART during pregnancy	TENLAM-E	64	1 (1.5%)	*p* = 0.48 X^2^ = 2.459
	Other regimens	13	1 (7.7%)	
	None	6	0	

*HAART* Highly Active Antiretroviral Therapy, *TENLAM-E* tenofovir-lamivudine-efavirenz, *HBV* hepatitis B virus; In bold are significant HBV prevalence

According to knowledge of maternal HBV status during pregnancy, children born from mothers without knowledge of HBV as compared to those from mothers who knew their HBV status: 2.9% vs. 0% respectively, *p* = 0.0097 (Table 2).

According to maternal history of ART, children born from mothers receiving ART consisting of TENLAM-E were appeared less likely to be infected with HBV as compared to those born from mother receiving other ART regimens: 1.5% (1/64) vs. 7.7% (1/13), *p* = 0.48; as shown in Table 2.

Additionally, the 2 HBV-positive children experienced exclusive breastfeeding, did not benefit from antiseptic bath at birth, had not received anti-HBV vaccination, and were from multiparous mothers, without any significant effect on HBV-positivity (Table 2).

### Performance of HBV rapid diagnosis tests

Due to reagent shortage, a total of just 47 participants were tested with both the two RDT and with EIA-HBsAg test Kit. The two (2) positive cases on EIA were reported as positive with HBSAg DiaSpot®rapid test (100% sensitivity) while only one was reported as positive with HBV 5 in 1, thus indicating a case of false negative result (50% sensitivity). EIA-HBsAg test Kit produced 45 negative results, which were all reported as negative with HBSAg DiaSpot®rapid test (100% specificity) while 44/45 were reported as negative with HBV 5 in 1 kit, thus indicating one case of false positive result (97.8% specificity) (Table 3).

**Table 3:** Performance of HBV RDTs with reference to EIA

	Diaspot		HBV-5	
	Positive	Negative	Positive	Negative
EIA (Gold standard)				
Positive	2	0	1	1
Negative	0	45	1	44
Total	2	45	2	45

*HBV-5* Hepatitis B 5 markers in one rapid diagnosis test, *EIA* Enzyme Immuno-Assay, *HBV* hepatitis B virus, *RDT* rapid diagnostic tests

With reference to EIA results, PPV of HBSAg DiaSpot® and HBV 5 in 1 test kit was respectively 100% (2/2) and. 50% (1/2). Regarding NPV, HBSAg DiaSpot®rapid test had a higher performance (100%) compared to HBV 5 in 1 kit (97.8%). Detailed results are shown in Table 3.

## Discussion

In RLS with a high burden of HIV and HBV, evidence favouring an easy integrated care of HIV/HBV are necessary to scale-up interventions towards meeting the global target of eliminating both HIV and HBV by 2030, especially for children who are generally among the most vulnerable [7, 13]. Achieving these goals require an understanding of the epidemiological burden, the risk factors involved, and knowledge on reliable HBV RDTs.

From our study participants, the sex distribution was similar (54.2% female, ratio F/M of 5/4),similar to a distribution found in a previous study in the same setting [13]. Though some studies found men to be slightly higher in proportion [21–23], the reported distribution between girls and boys in our study is within the range of birth rate proportions in the country. This therefore ensures a possible representativeness of our findings to the target population of CLHIV in Cameroon [24]. With a mean age of 8.7 years old, our findings are concordant with previous reports (mean age of 7.3 ± 3.6 years in Nigeria) in 201 6[21], thus ensuring comparability. However, age distribution was different from a previous study, due to differences in the primary aims and eligibility conditions (mean age of 26.6 and min-max: 6- 59 months) [13].

HBV-positivity was relative moderate (2.4%), and was similar to previous findings from the target populations in Cameroon (4.3%) [13], and in other countries (2% in Ethiopia [22], 1.6% in Democratic republic of Congo [25], 1.2% in Tanzania [26], 2.2% in Malawi [27], 3.3% in Thailand [28]). Compared to the highly endemic HBV among adult populations [15] or pregnant women (17, 5%) [18], the relatively moderate pediatric HBV prevalence is probably due to the wide paediatric coverage of anti-HBV vaccination in Cameroon [29]. This moderate prevalence of HBV in children could be partly attributed to maternal ART containing essentially TENLAM-E (77.1%), a regimen known to have molecules with antiviral activity (tenofovir and lamivudine) against HBV infection [7].

Regarding risk factors of HBV infection among these children, age 10–15 years appears with a higher (5%) but non-significant risk (*p* = 0.78) of HBV acquisition compared to younger ones (0%), as confirmed by previous studies [21, 22, 30]. Though non-significant, the presence of HBV only among in older children with advanced age, in a context of no reported sexual activity, suggests elow risk of HBV mother-to-child transmission and underscores the possibility of horizontal transmission of HBV(unsafe environment). Also, this may reflect the inability to achieve HBsAg clearance due to impaired immunity [31, 32]. Most importantly, the, hypothesis of low HBV prevalence or ART-tailoring HBV-infection seems to be the most prominent, owing to the fact that co-infected mothers with positive HBeAg, HBsAg and HBV DNA transmitted HBV to their children in Burkina-Faso [32].

Infants who experienced a vaginal delivery vs. Caesarean section are more at risk of HBV acquisition, as previously reported in China (RR = 2.20, 95% CI 1.02–4.74, *p* = 0.04) [33]. Of relevance, the two cases of positive HBV are from mothers with unknown HBsAg status during pregnancy, another factor significantly associated with HBV-positivity in CLHIV (*p* = 0.0097), due to no preventive or prophylactic measure undertaken against HBV during delivery. This calls for a systematic HBV testing of each pregnant woman with unknown HBV status in the labour room and the use of anti-HBV serum for infants born to HBsAg-positive mothers, as currently practised not only the USA [34].

Children from mothers receiving ART consisting of TENLAM-E appeared to have a slightly lower risk of HBV acquisition. Though not significant, this difference may reflect the ability of the aforementioned regimen (containing tenofovir and lamivudine) to control HBV; this observation merits further assessment in larger studies. Of note, feeding option and antiseptic bath did not have any effect on the risk of HBV acquisition, thus suggesting the effectiveness of protective breastfeeding demonstrated in the prevention of mother to child transmission [35–38], pending confirmatory findings on HBV as the amniotic fluid may contain HBV DNA [39]. HBV-positivity was also in the frame of no anti-HBV vaccination, supporting the benefit of universal vaccination of children [40], for ensuring a consistent decline of HBV (i.e. 88.5% decline with HBV vaccine coverage in USA) [41].

Regarding the diagnostic performance of the two HBV RDTs, DiaSpot®rapid test has excellent intrinsic performance (100% sensitivity, 100% specificity) and extrinsic performance (100% PPV and 100% NPV). However, the HBV 5 in 1 kit revealed poorer intrinsic performance (50% sensitivity, 97.8% specificity) and extrinsic performance (50% PPV and 97.8% NPV). These observations are highly concordant with previous findings on pooled analysis of HBV 5 in 1 kit showing sensitivity and specificity lower than the World Health Organisation (WHO) prequalified RDTs among people living with HIV (pooled sensitivity and specificity of 72.3 and 99.8% respectively) [42–47]. Thus, in a context of limited resources, Diaspot test should be prioritised for HBV testing on an individual living with HIV-infection [48]. The observed difference in sensitivities of both RDTs could be due to the fact that HBV variants are not effectively recognized by the antibodies coated on the test kit, leading to loss of or weakened epitope binding affinity [49].

The relative low-rate of HBV could also be due to events of occult HBV infection, generally characterised by a negative HBsAg and positive HBV DNA. This suggests further studies using molecular diagnostic platforms [4, 50]. Of note, vaccine-escape mutants within the ‘a’ determinant of the S genes are not genuinely recognized by conventional diagnostic tests as the wild type particles [50]. More so, hypothesis of occult hepatitis B in HIV-positive persons exposed to tenofovir- or lamivudine-containing ART regimens should be taken into consideration while explaining this low HBsAg prevalence and the reduced clinical sensitivity of the kit HBV-5 in 1[42]. A wider sampling and the use molecular assays would be more informative.

### Study limitations

Knowledge on the effective routes of HBV transmission, either vertical or horizontal, could not be delineated in the current study. Also, a thorough performance of both rapid tests under evaluation requires a higher sample size, which in our study was hindered by limited availability of financial resources. Further studies covering these epidemiological and diagnostic aspects would be relevant.

## Conclusion

Among paediatric populations living with HIV in an urban African setting, HBV-infection appears endemic at a moderate prevalence, suggesting a decreased burden possible attributed to preventives measures including the wide vaccine coverage. In this context, priority interventions towards mothers with unknown HBV-status and those practicing vaginal delivery (encouraging caesarean section for HBV-positive mothers) would strengthen current efforts in eliminating pediatric HBV in endemic countries. In such countries, coupled with limited resources, Diaspot test appears more reliable to rollout HBV-infection in the population of CLHIV and speed-up the global targets for HBV elimination.

## Data Availability

All the data on which the conclusions of the manuscript are drawn are duly presented in the main paper and related tables and figures.

## Abbreviations

ART: Antiretroviral therapy
DNA: Desoxyribo nucleotidic acid
EIA: Enzymes immuno assay
ELISA: Enzymes linked immunosorbent assay
HAART: Highly active antiretroviral therapy
HBeAg: Hepatitis B envelop antigen
HBsAg: Hepatitis B surface antigen
HBV-5: Hepatitis B 5 markers in one rapid diagnosis test
HIV: Human immunodeficiency virus,
IQR: Interquartile range,
NPV: Negative predictive value
PCR: Polymerase chain reaction
PPV: Positive predictive value
TENLAM-E: Tenofovir-lamivudine-efavirenz
WHO: World health organisation,
